# Complete Nucleotide Sequence Analysis of the Norovirus GII.4 Sydney Variant in South Korea

**DOI:** 10.1155/2015/374637

**Published:** 2015-01-19

**Authors:** Ji-Sun Park, Sung-Geun Lee, Ji-Young Jin, Han-Gil Cho, Weon-Hwa Jheong, Soon-Young Paik

**Affiliations:** ^1^Department of Microbiology, College of Medicine, The Catholic University of Korea, 222 Banpo-daero, Seocho-gu, Seoul 137-701, Republic of Korea; ^2^Korea Zoonosis Research Institute, Chonbuk National University, Iksan 570-390, Republic of Korea; ^3^Division of Virology, Gyeonggi Provincial Research Institute of Public Health and Environment, Suwon 440-290, Republic of Korea; ^4^Environmental Infrastructure Research Department, National Institute of Environmental Research, Incheon 404-708, Republic of Korea

## Abstract

Norovirus is the primary cause of acute gastroenteritis in individuals of all ages. In Australia, a new strain of norovirus (GII.4) was identified in March 2012, and this strain has spread rapidly around the world. In August 2012, this new GII.4 strain was identified in patients in South Korea. Therefore, to examine the characteristics of the epidemic norovirus GII.4 2012 variant in South Korea, we conducted KM272334 full-length genomic analysis. The genome of the gg-12-08-04 strain consisted of 7,558 bp and contained three open reading frame (ORF) composites throughout the whole genome: ORF1 (5,100 bp), ORF2 (1,623 bp), and ORF3 (807 bp). Phylogenetic analyses showed that gg-12-08-04 belonged to the GII.4 Sydney 2012 variant, sharing 98.92% nucleotide similarity with this variant strain. According to SimPlot analysis, the gg-12-08-04 strain was a recombinant strain with breakpoint at the ORF1/2 junction between Osaka 2007 and Apeldoorn 2008 strains. This study is the first report of the complete sequence of the GII.4 Sydney 2012 strain in South Korea. Therefore, this may represent the standard sequence of the norovirus GII.4 2012 variant in South Korea and could therefore be useful for the development of norovirus vaccines.

## 1. Introduction

Norovirus (NoV) is the most common cause of acute gastroenteritis in individuals of all age groups worldwide [1, 2]. NoV causes various diseases that are readily transmitted through the fecal-to-oral route, person-to-person contact, or contaminated food or water [3–6]. NoV has an incubation period of 12–24 h and presents with major symptoms typical of gastroenteritis, such as nausea, vomiting, diarrhea, abdominal pain, and fever. The symptoms usually appear for 1-2 days but can be fatal in rare cases [6].

NoV belongs to the family Caliciviridae, comprising single, positive-strand RNA genomes about 7.5 Kb in length. The viral genome is known to encode three open reading frames (ORFs): ORF1, ORF2, and ORF3 encode nonstructural proteins, VP1 major capsid proteins, and VP2 minor capsid proteins, respectively [7]. NoVs can be classified into five genogroups (I, II, III, IV, and V) according to the genetic makeup of the capsid protein and the RNA-dependent RNA polymerase (RdRp). Most human infections are caused by GI, GII, and GIV NoVs [8]. In the United States of America (USA), NoV has been shown to be a major cause of large-scale food poisoning [9], and the US Centers for Disease Control and Prevention (CDC) reported that NoV was detected in up to 96% of all viral gastroenteritis cases during the period from 1996 to 1997 [10]. Furthermore, the Korea Centers for Disease Control and Prevention (KCDC) confirmed that NoV was the cause of gastroenteritis in 1,046 patients who consumed contaminated water and food in 2013 [11]. Therefore, NoV is a major threat to human health worldwide, and the incidence of gastroenteritis is increasing rapidly.

Studies have shown that the GII.4 strain of NoV is becoming more prevalent in many countries around the world [12]. In addition, the GII.4 genotype has been found to exhibit a high mutation rate, with major variants emerging every 2-3 years [13, 14]. NoV GII.4 variant strains have been detected as follows: US 1995/96, Farmington Hills 2002, Hunter 2004, Yerseke 2006a, Den Haag 2006b, Osaka 2007, Apeldoorn 2008, and New Orleans 2009. Additionally, in March 2012, a new GII.4 NoV variant was found in Sydney, Australia [15]. Therefore, this new variant strain caused major episodes of food poisoning in many countries, including Hong Kong, Brazil, Japan, Italy, and Canada [16–20]. Unfortunately, despite the prevalence of NoV infections, no effective vaccine has been developed.

In South Korea, the NoV GII.4 Sydney variant was first detected in May 2012 [21]; moreover, few studies have described this new South Korean variant of the Sydney strain [22]. Therefore, in this study, we determined the complete genome sequence of the NoV GII.4 Sydney strain isolated from a stool sample of a patient with acute gastroenteritis in South Korea. The development of vaccines and the diagnosis of viral infections depend greatly on the elucidation of genome sequences. Our data are expected to provide important insights into the genetic characteristics of this virus, thereby facilitating the development of an effective NoV vaccine.

## 2. Materials and Methods

### 2.1. Specimen Collection

The stool sample was collected from a patient with symptoms of gastroenteritis at the Gyeonggi Institute of Health and Environment (GIHE, Gyeonggido, South Korea) in August 2012. GIHE confirmed the NoV genotype, and the specimen was sent to the Waterborne Virus Bank (WAVA, Seoul, South Korea) in May 2013. After the sample used for this study was confirmed to be the NoV GII.4 Sydney variant reported in a previous study [23], we received an additional sample from the WAVA and analyzed the complete nucleotide sequence of the GII.4 Sydney variant. The stool sample was stored at −70°C until analysis.

### 2.2. RNA Extraction

The stool sample was prepared as a 10% (w/v) suspension in phosphate-buffered saline (PBS; pH 7.2) and was centrifuged for 30 min at 13,000 rpm, at 4°C to extract RNA. Viral RNA was extracted from 140 *μ*L of supernatant using a QIAamp Viral RNA Mini Kit (Qiagen, Hilden, Germany), following the manufacturer's protocol. The extracted RNA was used as a template for reverse transcription polymerase chain reaction (RT-PCR).

### 2.3. RT-PCR Amplification

The full-length genome of NoV was amplified using eight pairs of previously designed primers and three pairs of newly designed primers (Table 1). The new primers were designed based on the published sequences of GII.4 Sydney variants of NoV. RT-PCR was performed with the primers shown in Table 1, and amplification was carried out using a Qiagen One-Step RT-PCR Kit (Qiagen) and an S1000TM Thermal Cycler (Bio-Rad, Singapore) for complete sequencing. The conditions for RT-PCR were as follows: reverse transcription at 50°C for 30 min, initial PCR activation at 95°C for 15 min, followed by 39 cycles of denaturation at 94°C for 1 min, annealing at 49–55°C for 1 min, and extension at 72°C for 1 min, followed by final extension at 72°C for 10 min. PCR products were analyzed by electrophoresis on 2% agarose gels containing ethidium bromide to confirm PCR amplification.

### 2.4. Cloning and Sequencing of the Complete Genome

The PCR products were purified from the 2% agarose gels with a HiYield Gel/PCR DNA Fragments Extraction Kit (RBC, Taipei, Taiwan). The purified products were cloned into the pGEM-T easy vector (Promega, Madison, WI, USA) and transformed into* Escherichia coli* DH5*α* competent cells (RBC) according to the manufacturer's instructions. Transformants were selected on Luria-Bertani (LB) agar (Duchefa, Haarlem, Netherlands) containing 50 mg/mL ampicillin, 0.1 mM isopropyl-*β*-d-thiogalactoside, and 40 mg/mL X-gal at 37°C for 16–18 h. Transformed clones were inoculated in LB Broth (Duchefa) and were subsequently cultured for 16–18 h in a shaking incubator (37°C, 200 rpm; IS-971R, Jeiotech, Daejeon, South Korea). Plasmid DNA was purified using a HiYield Plasmid Mini Kit (RBC), and plasmids were sent to Cosmogenetech Co. Ltd. (Seoul, South Korea) for sequencing. The complete nucleotide sequence of the gg-12-08-04 NoV strain was deposited in GenBank under accession number KM272334.

### 2.5. Phylogenetic Analysis

Multiple sequence alignments were performed with Clustal W using Molecular Evolutionary Genetics Analysis software (MEGA version 6.0) [24]. Phylogenetic trees were created using the neighbor-joining method with a Kimura two-parameter model in MEGA [25], and branch support was calculated based on 1,000 bootstrap replicates. Based on NoV GII.4 genotyping, the complete genome sequences were collected using a BLAST search of the National Center for Biotechnology Information (NCBI).

### 2.6. Recombination Analysis

Whole genome sequences of two reference strains and the gg-12-08-04 strain were analyzed using SimPlot software (Ver 3.5.1) to confirm putative recombination events [26]. The SimPlot analysis was carried out using the Kimura two-parameter distance model with a window size of 200 bp and a step size of 20 bp with the gap strip on. Results were visualized as percent similarity.

### 2.7. Ethics Statement

The sample used in this study was approved by the Catholic Medical Center Office of Human Research Protection Program (CMC OHRP) of South Korea (approval number MC14SASI0069). Written informed consent was provided by the patient with symptoms of gastroenteritis, and this information has been kept on file at the GIHE and WAVA.

## 3. Results and Discussion

**Figure 1:**
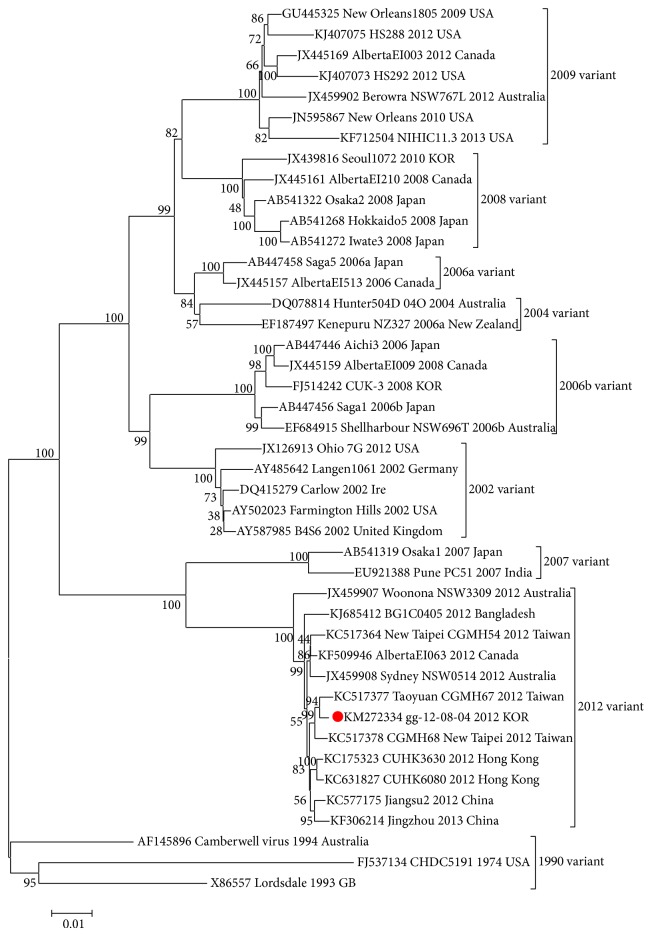
Phylogenetic trees based on nucleotide sequences of the NoV GII.4 strain for full genomic sequences. The trees were constructed by the neighbor-joining method. Numbers on each branch indicate the bootstrap values obtained from 1,000 replicates. The following isolates were analyzed (accession numbers/strain named/collection year/nation/region). The gg-12-08-04 is indicated by a red circle. Reference sequences were as follows: Lordsdale (X86557), Camberwell (AF145896), CHDC5191 (FJ537134), Ohio 7G (JX126913), Langen1061 (AY485642), B4S6 (AY587985), Carlow (DQ415279), Farmington Hills (AY502023), Hunter504D (DQ078814), Saga5 (AB447458), NZ327 (EF187497), AlbertaEI513 (JX445157), CUK-3 (FJ514242), Saga1 (AB447456), AlbertaEI009 (JX445159), Aichi3 (AB447446), NSW696T (EF684915), Osaka1 (AB541319), PC51 (EU921388), AlbertaEI210 (JX445161), Seoul1072 (JX439816), Osaka2 (AB541322), Iwate3 (AB541272), Hokkaido5 (AB541268), HS288 (KJ407075), HS292 (KJ407073), NIHIC11.3 (KF712504), New Orleans (JN595867), AlbertaEI003 (JX445169), New Orleans 1805 (GU445325), NSW767L (JX459902), Jingzhou (KF306214), AlbertaEI063 (KF509946), BG1C0405 (KJ685412), CUHK6080 (KC631827), CUHK3630 (KC175323), Sydney NSW0514 (JX459908), Woonona NSW3309 (JX459907), CGMH54 (KX517364), Jiangsu2 (KC577175), CGMH68 (KC517378), and CGMH67 (KC517377).

In this study, we analyzed the nucleotide sequence of the entire genome of the NoV 2012 strain isolated in South Korea; the viral strain was named gg-12-08-04. The complete genome sequence of the gg-12-08-04 strain was 7,558 nucleotides (nt) in length and contained three ORFs: ORF1 (5,100 nt), ORF2 (1,623 nt), and ORF3 (807 nt). The isolated strain gg-12-08-04 shared high sequence homology with NoV GII-4. BLAST results using the complete gg-12-08-04 genome sequence as the query showed that the sequence with the highest similarity (query coverage = 99%) belonged to CUHK3630 (GenBank accession number KC175323), and the sequence similarities with other NoV strains ranged from 85.95% to 99.15%. Phylogenetic analysis was performed to evaluate the genetic relationships among samples and representative GII.4 strains. As shown in the tree, the whole genome sequences of the gg-12-08-04 strain were classified as a 2012 variant. The full-length nucleotide sequences shared highest identity (99.27% and 99.47%, resp.) with the CUHK3630 strain (Figure 1). The gg-12-08-04 strain shared 98.92% nucleotide similarity and 97.90% amino acid similarity over the full-length sequence with the original Sydney 2012 strain (GenBank accession number JX459908; Figure 1). Also, 12, 7, and 5 amino acids within ORF1, ORF2, and ORF3, respectively, differed between gg-12-08-04 and Sydney strains. Additionally, we identified three novel sites within ORF1 (371, 1,004, and 1,055) and five novel sites within ORF2 (95, 148, 264, 319, and 464) (data not shown).

Amino acid sequence changes within the surface-exposed subdomain P2 region correlate with the emergence of new epidemic strains through changes in the antigenic structure [27]. Importantly, the emergence of novel NoVs is associated with mutations in five evolving blockade epitopes (A–E) within the capsid P2 domain [28]. Compared with the A–E epitopes of nine representative reference strains, the A–E epitopes of the gg-12-08-04 strain were different from those of the 2009 New Orleans strain, but the same as those of the 2012 Sydney strain; however, three amino acid substitutions in the P2 region were identified in AA310 (aspartic acid → asparagine), AA319 (alanine → valine), and AA373 (arginine → histidine). Additionally, the asparagine residue found at amino acid position 373 was replaced by arginine in the Sydney strain and histidine in the gg-12-08-04 strain (Table 2).

**Figure 2:**
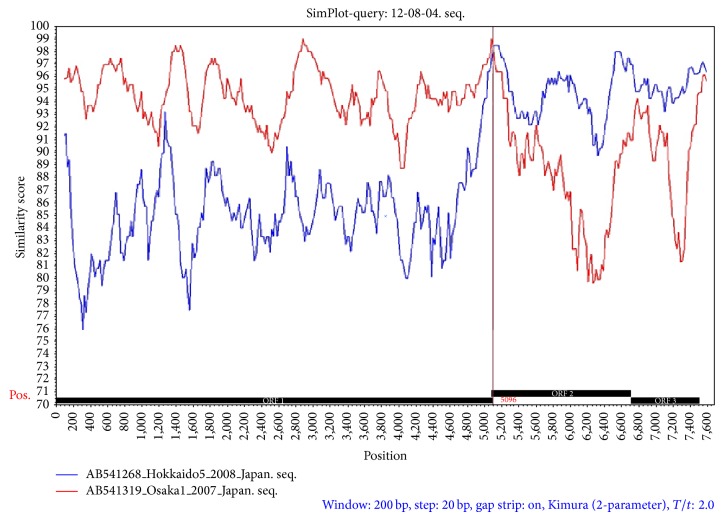
SimPlot analysis of full sequences showed the similarities and the breakpoint among gg-12-08-04, Osaka 2007, and Hokkaido5 2008 strains. The SimPlot settings were as follows: window, 200 bp; step, 20 bp; gap string, on. The vertical axis, horizontal axis, and dashed line indicate the nucleotide similarity percentage, nucleotide position, and putative recombination breakpoint, respectively.

Recombination events resulting in breakpoints within the GII.4 strains have occurred frequently between ORF1/2 and ORF2/3 and within ORF2; moreover, the ORF1/2 junction has been identified as a site of recombination in the NoV genome [29, 30]. Several novel recombinant NoV strains of 2012 variants have recently been reported. Recombination within the ORF1/ORF2 junction has been reported in Italy, South Africa, and Denmark [31–33]. According to previous studies [29], the ORF1 region of the Sydney 2012 strain was derived from the Osaka 2007 strain (GenBank accession number AB541319), and the ORF2 and ORF3 regions were derived from the Apeldoorn 2008 strain (GenBank accession number AB541268). The putative recombination breakpoint was located at nucleotide position 5,096 within the ORF1/2 junction region, and this region shared 98.23% and 98.24% nucleotide sequence identity with the Osaka 2007 and Hokkaido5 2008 strains, respectively. The gg-12-08-04 strain showed 94.61% similarity with the Osaka 2007 strain and 85.28% similarity with the Hokkaido5 2008 strain at nucleotide positions 101–5,096. At nucleotide positions 5,096–7,558, gg-12-08-04 exhibited 88.94% and 94.97% nucleotide sequence similarities with the Osaka 2007 and Hokkaido5 2008 strains, respectively (Figure 2).

## 4. Conclusions

Because the Sydney 2012 variant has rapidly diversified within the population, as shown in recent studies [34], an immunological study of NoV variants is needed to examine the range of the outbreak. In this study, we provided evidence of the rapid spread of the Sydney 2012 strain, which was identified in South Korea within 5 months of its emergence. Similar global trends were observed in Japan and Canada [18, 20].

In conclusion, we reported the first complete genome sequence of the GII.4 Sydney 2012 variant in South Korea, and we expect that these data will be useful for the prevention of NoV and analysis of NoV gene function. These data will also contribute to the design of NoV vaccines based on the complete sequence of the 2012 variant in South Korea.

## Figures and Tables

**Table 1:** Information of primers used in this study.

Region	Primers	Sequence (5′-3′)	Polarity	Position^a^	Reference
ORF1-ORF2	GII-F1M	GGG AGG GCG ATC GCA ATC T	+	5049–5067	
	GII-R1M	CCR CCI GCA TRI CCR TTR TAC AT	−	5367–5389	

ORF1	ORF1-1F	GTG AAT GAA GAT GGC GTC TA	+	1–20	[23]
	ORF1-1R	AGT CTT GGT AGG GCC TAA AG	−	697–716	
	ORF1-2F	GGC TAA GCA GGA GAA TGA TTC A	+	660–681	In this study
	ORF1-2R	GAG AGT TGA TTG TGC CCA CA	−	1375–1394	
	ORF1-3F	CCA AGT CTG CTT CAC CTG AC	+	1353–1372	[23]
	ORF1-3R	GGG TGT TTC CGT TCT TGT C	−	1976–1994	
	ORF1-4F	ACT GTC ATT GGC TCC ACA G	+	1948–1966	In this study
	ORF1-4R	TGT GTG CTT CTT GCC ACG	−	2657–2674	
	ORF1-5F	GAC GAC ATC AAA ACT GAG GGC	+	2612–2632	
	ORF1-5R	TCT TGA TGA GCA GTG TGG C	−	3275–3293	
	ORF1-6F	GGC ATG ATC TTG GAA GAA GG	+	3236–3255	[23]
	ORF1-6R	ATG CTT GCG CGA ATG ACC	−	3882–3899	
	ORF1-7F	GGC TGC CAA GAA AAC CAT C	+	3823–3841	
	ORF1-7R	ACC TCA GAA AGT GCA CAG AG	−	4523–4542	
	ORF1-8F	CCA ATG GAA TTC CAT CGC CC	+	4489–4508	
	ORF1-8R	CGA CGC CAT CTT CAT TCA CA	−	5080–5099	

ORF2	ORF2-F1	AAG AGC CAA TGT TCA GAT GG	+	5004–5023	[22]
	ORF2-R1	CTC TGA AGG TGC AGA TGT T	−	5928–5946	
	ORF2-F2	AAC ATC TGC ACC TTC AGA G	+	5928–5946	
	ORF2-R2	GAA GCC TGT TGT AGA TTG CT	−	6854–6873	

ORF3	ORF 3F	ATG GCT GGA GCT TTC TTT GCT	+	6704–6724	
	ORF 3R	AAA GAC ACT AAA GAA AGG AAA GAT	−	7532–7555	

^a^Positions are indicated relative to the Lordsdale strain (GenBank accession number X86557).

**Table 2:** Amino acid differences in the hypervariable domain of ORF2.

GII.4 variant strains	Epitopes															
	A						B		C		D			E		
	294	296	297	298	368	372	333	382	340	376	393	394	395	407	412	413
X86557 Lordsdale																
1990 variant	A	S	H	D	T	N	L	K	A	Q	D	—	—	N	T	G
AY502023 Farmington Hills																
2002 variant	*·*	T	*·*	N	N	*·*	M	*·*	G	E	N	G	T	S	*·*	*·*
DQ078814 Hunter504D																
2004 variant	*·*	T	Q	N	S	S	V	R	R	E	S	T	T	D	D	S
AB447458 Saga5																
2006a variant	*·*	T	Q	E	S	S	V	R	R	E	G	T	T	*·*	D	S
AB447456 Saga1																
2006b variant	*·*	*·*	R	N	S	E	*·*	*·*	G	E	S	T	T	S	N	V
AB541319 Osaka1																
2007 variant	*·*	*·*	R	N	A	D	M	*·*	S	E	S	T	T	*·*	*·*	*·*
AB541268 Hokkaido5 2008 Japan																
2008 variant	T	*·*	R	N	A	D	V	*·*	*·*	D	D	T	A	S	N	S
GU445325 New Orleans1805																
2009 variant	P	*·*	R	N	A	D	V	*·*	T	E	S	T	T	S	N	I
JX459908 Sydney NSW0514																
2012 variant	**T**	***·***	**R**	**N**	**E**	**D**	**V**	***·***	**T**	**E**	**G**	**T**	***·***	**S**	**N**	**T**
KM272334 gg-12-08-04	**T** ^*^	***·***	**R** ^*^	**N** ^*^	**E** ^*^	**D** ^*^	**V** ^*^	***·***	**T** ^*^	**E** ^*^	**G** ^*^	**T** ^*^	***·***	**S** ^*^	**N** ^*^	**T** ^*^
